# Why does aging anxiety emerge? A study on the influence of socioeconomic status

**DOI:** 10.3389/fpsyg.2025.1602284

**Published:** 2025-08-22

**Authors:** Shuchao Yang, Daoshun Ge

**Affiliations:** ^1^Center for Faculty Development, Guangdong University of Finance and Economics, Guangzhou, Guangdong, China; ^2^International Academy of Red Cross and Red Crescent, Soochow University, Suzhou, Jiangsu, China

**Keywords:** socioeconomic status, stressors, perceptions of aging, aging anxiety, urban–rural structures

## Abstract

Aging anxiety constitutes a pressing practical issue impacting active aging and healthy aging of the population. Existing theories on aging anxiety predominantly analyze its intrinsic causes through psychosocial perspectives such as perception and identity formation, yet insufficient attention has been paid to structural factors like socioeconomic status that may influence aging anxiety. Current research findings regarding the impact of socioeconomic status on aging anxiety exhibit inconsistencies, while the analysis of underlying mechanisms requires further refinement. This study integrates the Fundamental Causes Theory of health and the Stress Process Theory, utilizing data from the Chinese General Social Survey (CGSS) to investigate the mechanisms through which socioeconomic status affects aging anxiety. The research reveals that socioeconomic status influences individual aging anxiety both directly and indirectly through mediating mechanisms involving anxiety stressors and perceptions of aging. Specifically, improvements in objective socioeconomic status indicators (education and income) and positive expectations regarding subjective socioeconomic status significantly alleviate aging anxiety. Higher educational attainment and income levels reduce individuals’ probability of encountering practical and anticipatory aging-related stressors such as illness and caregiving needs, while fostering more positive perceptions of aging, thereby diminishing aging anxiety. The influence of socioeconomic status on aging anxiety is moderated by regional urbanization levels and urban–rural structures. The anxiety-reducing effect of educational attainment strengthens in regions with higher urbanization levels. Rural residents experience stronger aging anxiety than urban residents, and increased participation in social insurance can effectively alleviate this anxiety among rural residents. This study provides valuable references for cultural interventions and policy-making aimed at alleviating aging anxiety.

## 1 Introduction

With the development and transformation of the economy and society, China is facing an increasingly severe challenge of population aging, characterized by a large elderly population base and a rapid pace of aging. According to the Statistical Communiqué of the National Bureau of Statistics of China, by the end of 2023, China’s population aged 60 and above reached 296.97 million, accounting for 21.1% of the total population. Among them, the population aged 65 and above reached 216.76 million, representing 15.4% of the total population. China’s average life expectancy reached 78.6 years in 2023. As life expectancy continues to rise, adapting to aging has become a common challenge that most individuals must confront. However, persistent age discrimination and stereotypes against the elderly in society have, to some extent, hindered the progress toward healthy aging at the societal level. Against this backdrop, research on people’s attitudes toward aging and their influencing factors holds significant practical significance.

Existing research demonstrates that negative views or beliefs about aging adversely affect the mental health (Levy et al., 2008) and subjective well-being (Faudzi et al., 2020) of older adults and middle-aged individuals (Yun and Lachman, 2006). Anxiety about aging can lead to issues such as death anxiety and depression (Benton et al., 2007). Fear of aging-related diseases and elderly loneliness may cause people to anticipate a shorter ideal life expectancy (Rupprecht et al., 2022). Anxiety is not merely an individual psychological response; it is also a routinized social practice (Jackson and Everts, 2010) and a collective sentiment (Rebughini, 2021). If left unaddressed, individual aging anxiety may spread (Wang, 2024) and transform into a pervasive societal mood (Zhang and Li, 2024), adversely impacting active healthy aging and socioeconomic development.

Existing research on aging anxiety predominantly analyzes factors such as gender, group identity, and intergroup contact. However, aging anxiety is not merely an issue confined to specific groups or identities; it necessitates exploration of influencing factors at a broader social structural level. Concurrently, current studies require strengthening in analyzing the causal mechanisms between influencing factors like socioeconomic status and aging anxiety. Empirical research exhibits certain contradictions in conclusions regarding the relationship between socioeconomic status factors (e.g., income, education) and aging anxiety.

Experiences of living in different environments and exposure to diverse cultures influence older adults’ perceptions and subjective experiences of aging. Given that Asian cultures are more oriented toward interdependence and collectivism, people may hold friendlier views and attitudes toward the aging process and the elderly population (Schwartz, 2006). Research on aging anxiety in China, as an Asian nation, remains relatively limited. This study seeks to integrate the Fundamental Cause Theory and the Stress Process Theory to analyze the mechanisms through which socioeconomic status affects aging anxiety, while also examining the impact of structural social factors such as urban–rural disparities and marketization levels on aging anxiety. The aim is to enrich theoretical knowledge about aging anxiety and provide references for elderly policy formulation and elderly support practices.

## 2 Literature review and research hypotheses

### 2.1 Aging anxiety and its doctrinal explanation

Aging anxiety is defined as people’s concerns about getting older, such as fears of declining health and physical functioning, financial problems, cognitive decline, changes in appearance, and loss of socialization, and it is also known as gerascophobia, fear of aging, and fear of growing old (Lynch, 2000). Anxiety of aging describes the negative feelings and fears associated with growing older, including physical, psychological, social, and transpersonal losses. Fears related to physical losses include changes in health and functional ability, anxiety about psychological losses include fear of dependence, loss of personal control, cognitive decline, and low life satisfaction, and concerns about social losses focus on interpersonal relationships, such as interactions with others quantity and quality, but also financial problems or lack of employment. Fears associated with transpersonal loss are related to how one faces death and how one assesses the meaning of life (Lasher and Faulkender, 1993). Aging anxiety can be understood as a state of anxiety resulting from the fear of anticipated threats associated with the aging process, and these anticipated threats subsequently become potential sources of aging anxiety (Watkins et al., 1998).

Research related to aging anxiety is reflected at the macro, meso, and micro levels. At the macro level, culture and values are often recognized as important factors in the aging process. It indicates that in more collectivism-oriented cultures such as those in East Asia, societies tend to hold more positive views on aging (O'Brien et al., 2017). In contrast, Western societies often prioritize youthfulness and maintain more negative perceptions of aging and older adults (North and Fiske, 2015). The opposite view has also been found, with countries in Asia, the Middle East, and Sub-Saharan Africa having significantly more negative views of older people than Western countries (Peterson and Ralston, 2017).

At the meso level, social institutions and social relationships have an impact on aging anxiety. Aging anxiety may be shaped by an individual’s attachment to various institutions, and marriage, family, and work can have an impact on an individual’s aging anxiety. Marriage not only protects physical and mental health, but may also act as a source of support in the face of illness, reducing anxiety about future declines in health, with divorced or separated women reporting more aging anxiety (Simon, 2002). Paid work may reduce concerns about declining health by providing greater financial security and health security at retirement age (Barrett and Robbins, 2008). The presence of supportive relationships with spouses and other family members may prevent some sources of anxiety, with the central role of the family in providing instrumental support in later life, and deteriorating relationships with spouses or partners and other family members may exacerbate anxiety about health decline (Barrett and Lynch, 1999). Social relationships are an important determinant of anxiety about aging, and better social relationships indirectly influence our anxiety about aging by enhancing our anticipation of our later life and our perception of our ability to cope with future challenges, generating a certain sense of security. At the same time, social relationships provide reference points for our self-reflexive appraisals and our experiences that may influence aging anxiety by shaping our self-appraisal. Friends of the same age and gender as ourselves may provide a frame of reference and emotional support for coping with age-related physical changes (McPherson et al., 2001), and the more stressful the relationship with a spouse/friend or the less support from friends of support the less likely one is to be at greater risk for aging anxiety (Barrett and Toothman, 2018).

Micro-analysis at the psychosocial and cognitive level plays an important role in the study of aging anxiety. Psychosocial theories such as Social Identity Theory, Terror Management Theory, Social Clock Theory, Double Standard Theory of Aging, and Stereotype Embodiment have explained aging anxiety. Terror Management Theory (TMT) suggests that aging anxiety is rooted in the fear of death and that aging, as the most direct reminder of death, causes anxiety. Physical signs of aging, such as declining beauty and health, contribute to the fear of aging (Martens et al., 2004), which is amplified by young people trying to stay away from older people to avoid death. According to Social identity theory (SIT), self-categorization and social comparison may help to explain aging anxiety. Based on social comparison, members of an internal group view themselves more positively and members of an external group more negatively, and in youth-oriented societies there may be negative attitudes toward older adults (Momtaz et al., 2013). Group identity and group motivation in young people are significant predictors of aging anxiety and ageism (Taşdemir, 2020), and moving from a younger to an older group can lead to anxiety. Social clock theory (SCT) describes society’s age-graded expectations for goals such as marriage and childbearing, and the fear of getting older can arise if people fail to accomplish important life tasks (Rook et al., 1989). According to the Double standard of aging theory (DSA), men are valued for their accomplishments, which increase with age, while women are valued for their appearance, which decreases with age. The loss of status, such as attractiveness and fertility, that accompanies aging can lead to aging anxiety in women (Barrett and Robbins, 2008).

Several microscopic mechanisms can also influence aging anxiety. Current health problems, as well as less favorable perceptions of one’s health and recent physiological changes, may increase anxiety about future health. Although some studies have shown that better health is associated with less worry about aging, this may vary depending on the source of anxiety and health indicators (Lynch, 2000). One study found a correlation between an individual’s personality traits and aging anxiety, with a positive correlation between neuroticism and overall anxiety about aging. Because of the negative correlation with personality traits such as extraversion, there is some stability in aging anxiety as a result (Harris and Dollinger, 2003). Self-efficacy, perceived health status, and religious beliefs may also influence aging anxiety in middle-aged women (Jung and Oh, 2016).

Existing theoretical and empirical studies have examined the sources and mechanisms of aging anxiety across cultural, psychological, and micro-level dimensions. Theoretical explanations often emphasize intrinsic psychosocial factors such as perception and identity formation in driving the development of aging anxiety (Momtaz et al., 2021). However, from the perspective of the Fundamental Cause Theory (Link and Phelan, 1995), socioeconomic status serves as a foundational determinant of health. When aging anxiety is contextualized within the broader framework of mental health, critical questions emerge: What shapes individuals’ perceptions and the formation of social identities? Do structural factors like socioeconomic status directly influence aging anxiety, and do they mediate the development of individuals’ perceptions and social identities?

Although some Chinese researchers have pointed out the existence of aging-related fears and discrimination in traditional beliefs (Chen and Wu, 2024), empirical analyses on aging anxiety remain relatively limited. This research will explore whether the impact of socioeconomic status on aging anxiety is moderated by urban–rural disparities and marketization levels, thereby analyzing the mechanisms of aging anxiety formation through a macro-structural societal framework. The findings seek to advance theoretical insights into aging anxiety and inform policies and practices aimed at fostering equitable aging experiences in a rapidly transforming society.

### 2.2 Socioeconomic status and aging anxiety

Research on the relationship between socioeconomic status (SES) and aging anxiety has gained scholarly attention. However, existing findings exhibit contradictions, and the mechanisms linking socioeconomic status to aging anxiety remain inadequately theorized and fragmented.

Socioeconomic status comprises objective socioeconomic status and subjective socioeconomic status. Objective SES is typically measured through indicators such as income, education, and occupation. Subjective socioeconomic status refers to individuals’ perceptions of their position within the socioeconomic hierarchy (Singh-Manoux et al., 2003). Frequent discrepancies exist between objective and subjective SES. Subjective status identification is context-dependent; while influenced by objective SES, it demonstrates only moderate correlation with objective measures (Kraus and Keltner, 2009; Huang, 2020). Objective status indicators shape personal expectations and self-perceptions of social standing. Through social comparison processes, individuals integrate objective social positions with personal characteristics, internalizing them as subjective reality (Singh-Manoux et al., 2003). Simultaneously, this subjective perception functions as a socio-psychological factor that operates independently beyond its objective foundations (Cohen et al., 2008). The impacts of both objective and subjective socioeconomic status on mental health outcomes—particularly aging anxiety—have garnered extensive scholarly investigation.

Cumulative disadvantage theory suggests that a lifetime of low socioeconomic status accelerates the onset of unfavorable conditions and events (O'Rand, 1996). Concerns about the ability to cope with loss, support oneself financially, and the ability to receive adequate health care in old age may be particularly acute for those without financial resources (Dannefer, 2003), leading them to anticipate a problematic old age. One study found a negative correlation between various measures of socioeconomic status and anxiety about physical health in old age (Williams and Collins, 1995), with having more socioeconomic resources—such as education, household income, or relative contribution to household income—is associated with lower levels of anxiety about declining health (Phelan and Link, 2013). Some studies using comprehensive measures of aging anxiety have found that socioeconomic advantages represented by higher education and income predict lower anxiety (Abramson and Silverstein, 2004; Yan et al., 2011).

However, other studies have found that disadvantaged status (e.g., older adults, people with disabilities) does not necessarily produce higher anxiety about aging relative to groups with advantaged socioeconomic status, which may be related to the type of aging anxiety. This is explained by the crisis competence perspective of aging, which suggests that some disadvantaged groups learn strategies for adapting to aging through early life challenges (Pound et al., 1998), may have little or no anxiety about age-related physical changes, and that aging anxiety this pales in comparison to more urgent daily life difficulties (Dillaway et al., 2008). Other research has revealed social constructions of age change and individual strategies for ‘reconciling’ with these changes, which may protect vulnerable groups from aging anxiety (Agee, 2010), leading to a sense of well-being.

Research also indicates that the relationship between socioeconomic status and aging anxiety varies depending on the content of the aging anxiety, with women having less anxiety over declining economic status, health, or attractiveness the more economically independent they are, but more anxiety over reproductive aging instead (Barrett and Toothman, 2018).

Based on the above analysis, the research hypothesis is proposed:

*Hypothesis* 1.1: Higher levels of personal income are associated with higher levels of aging anxiety.

*Hypothesis* 1.2: Higher levels of personal income are associated with lower levels of aging anxiety.

Individuals’ subjective status identification is not merely determined by their current socioeconomic position—such as education or income—but is significantly influenced by the relative mobility of their socioeconomic standing (Liu, 2002). Indeed, perceptions of social mobility may exert a stronger influence than static socioeconomic status itself (Chen et al., 2019). Research indicates that those with higher subjective status tend to possess enhanced capacities for stress resilience and emotional regulation. These advantages permeate daily behaviors and psychological states, ultimately benefiting physical health (Demakakos et al., 2008). Studies focusing on adults over 60 reveal that subjective socioeconomic status more effectively predicts perceptions of aging: higher subjective status correlates with more positive attitudes toward aging and diminished awareness of age-related losses (English et al., 2019).

From a static perspective, both objective and subjective socioeconomic status shape individual health and psychosocial processes. Yet through a dynamic lens, socioeconomic status is inherently fluid. Expectations of future mobility moderate current status, generating divergent psychological consequences. When individuals anticipate upward mobility within their perceived status tier, they preemptively adopt cognitive dispositions of higher strata, manifesting stronger self-control efficacy. Heightened upward mobility expectations amplify positive subjective perceptions of self, others, and society (Zhou, 2022)—potentially fostering constructive perceptions of aging and thereby reducing aging anxiety. Addressing the prevalent focus on current status assessments in existing research, Kuball and Jahn (2024) employed latent profile analysis to demonstrate that older adults with initially low subjective status or perceived downward mobility exhibited the most severe aging anxiety and negative affect.

Building upon this analysis, we propose the following research hypothesis:

*Hypothesis* 1.3: Individuals’ subjective expectations of rising socioeconomic status will have a dampening effect on aging anxiety.

In terms of education level and aging anxiety, one study found that lower levels of education predicted less anxiety about declining attractiveness with age (Barrett and Toothman, 2018). Higher levels of education are positively associated with higher levels of mortality concern (Waskel, 1995). However, it has also been found that respondents with low levels of education and low incomes report greater aging anxiety than respondents with higher levels of education and higher income categories (Yan et al., 2011). Education can reduce death anxiety, and higher levels of education can increase a person’s autonomy (Henderson, 1990), further reducing death and aging anxiety. One study found that education had no effect on aging anxiety in the youngest age group, but had a significant negative effect on aging anxiety in older adults (Lynch, 2000). Based on the above analysis, the research hypothesis is proposed:

*Hypothesis* 2.1: Higher levels of education are associated with lower levels of aging anxiety.

*Hypothesis* 2.2: Higher levels of education are associated with higher levels of aging anxiety.

Pearlin’s stress process theory elucidates the causal relationship between social structural arrangements and mental health (Pearlin and Bierman, 2013), explaining why the likelihood of people experiencing problematic mental, emotional, and behavioral states depends on where they are in the hierarchical system and the extent to which they are engaged in social institutions and relationships (Pearlin, 1989). Pearlin’s stress process theory provides ideas for analyzing the intermediate mechanisms of action from socioeconomic status to aging anxiety. Stress process theory states that the different socio-economic statuses of individuals determine the differences in the sources of their stress, with those in disadvantaged positions being the most likely to be exposed to and affected by unfavorable stressors. Stressors are mediated or mediated by resources such as an individual’s control and social support to produce stressful consequences such as mental health. The main representatives of stressors are negative events and daily pressures, as well as Anticipatory Stressors. Of these, Anticipatory Stressors do not exist as realities but are seen as having the potential to become realities, e.g., when difficulties befall other people, people may increasingly expect that their own lives will be affected by the same threatening circumstances. On a wider scale, fluctuations in economic conditions associated with society as a whole will lead to the awakening of relevant Anticipatory Stressors, and those who suffer directly from the financial stresses associated with economic downturns may suffer detrimental mental health consequences (Zivin et al., 2011), When economic hardship begins affecting individuals within one’s relational networks and becomes a routine theme in media coverage, the misfortunes of others may contribute to anxiety and concern among those not yet directly experiencing such pressures. Secondary stressors can be derived within the stressor or trigger other stressors that can spread laterally between roles, e.g., Children’s difficulties or adversity may become a stressor for parents, and caring for an elderly person with a medical condition or other family members may create perceptions of role overload or role capture, which in turn affects socialization or life, and negative perceptions of aging (Aneshensel, 2015). Based on the above analysis, it is proposed:

*Hypothesis* 3: Individuals’ socioeconomic status can influence individual aging anxiety through the mediating role of aging anxiety stressors.

Many approach aging with fear and anxiety, for which ageist attitudes and stereotypes serve as significant contributors. Crucially, ageism may trigger anxiety in individuals of any age (Cummings et al., 2000). Nelson (2005) study noted that prejudice against aging is positively associated with aging anxiety and that ageism may be linked to aging anxiety through stereotypes and poor attitudes toward aging increasing young people’s fear of aging.

Beyond influencing societal behaviors toward older adults, care management approaches, and aging environments, stereotypes about old age also shape individuals’ personal experiences of aging (Ramírez and Palacios-Espinosa, 2016). Age stereotypes become embodied through cultural assimilation (Levy, 2009), impacting expectations regarding retirement, health, and mortality. This internalization fosters negative attitudes toward aging (Ramírez et al., 2019), which may subsequently trigger gerontophobia (fear of aging; Brunton and Scott, 2015).

Aging anxiety may also stem from deficient factual knowledge about the aging process. Cummings et al. (2000) research demonstrates that accurate understanding of aging correlates inversely with aging anxiety—precise comprehension of aging realities potentially alleviates future-oriented fears.

The Contact Hypothesis posits that both knowledge acquisition about aging and intergenerational contact reduce ageism against older adults (Barnett and Adams, 2018), thereby modulating aging anxiety (Allan and Johnson, 2008).

Ageism, aging stereotypes, and knowledge about aging reflect societal and individual cognition and attitudes toward old age. Concurrently, the relationship between socioeconomic status (SES) and individual cognition/social attitudes constitutes a significant scholarly concern. Research indicates that due to contextual cognitive orientation, individuals from lower social classes tend toward external attributions for life events, with long-term lower-class positioning heightening threat sensitivity (Manstead, 2018). Socioeconomically disadvantaged groups may develop constrained expectations regarding aging risk management due to relative scarcity of material and healthcare resources. Conversely, higher-SES groups likely experience reduced aging anxiety through enhanced perceived control over later life, afforded by greater access to resources (e.g., pensions, private insurance). Empirical work reveals divergent life outlooks among urban residents across income strata: middle-to-high-income groups exhibit more positive life attitudes compared to low-income counterparts (Lin et al., 2015). Crucially, subjective class consciousness significantly influences individual cognition, attitudes, and emotional states—lower subjective social class correlates with heightened insecurity and anxiety (Zhu and Zhu, 2015). Those perceiving themselves as middle or upper-middle class emerge as core bearers of positive social mindsets, demonstrating greater security, life satisfaction, and positive affect alongside diminished negative emotions (Wang, 2018). Based on the above analysis, the following is proposed:

*Hypothesis* 4: Individuals’ socioeconomic status may influence individuals’ aging anxiety through the mediating role of perceptions of aging.

**Figure 1:**
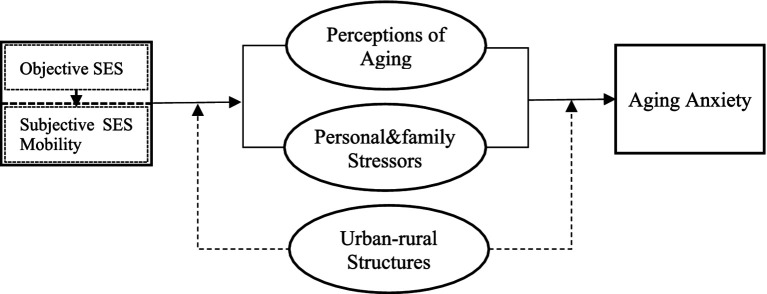
The impact and mechanisms of socioeconomic status on aging anxiety.

Victor Nee’s theory of market transformation points out that the market-oriented transformation after the reform and opening up has led to changes in China’s resource allocation mode, with the market’s position in resource allocation rising and the political capital’s position declining (Nee, 1989). With the improvement of marketization level, the return on personal political capital decreases, while the return on personal human capital increases. The return referred to by Victor Nee here mainly refers to economic returns, while the market-oriented transformation is accompanied by China’s promotion of institutional reforms in various aspects such as the economy, society, and culture with market-oriented ideas, which may have multidimensional impacts on individuals’ lives. Therefore, it is necessary to reevaluate the impact of marketization on diversified returns and well-being (Song and Xie, 2014). Empirical studies also indicate that elevated individual occupational status and increased regional marketization index can reduce depression levels; however, the impact of occupational status on depression is moderated by regional marketization levels. Paradoxically, individuals with high occupational status residing in highly marketized cities may experience an increase rather than a decrease in depression levels (Xu et al., 2023). Inspired by this, this article incorporates aging anxiety into the category of individual health and well-being, believing that it belongs to the return of human capital and other capital of individuals. It is assumed that the role of social and economic status indicators such as education and income on aging anxiety will be influenced by the level of marketization in the region where the individual is located. The higher the level of marketization in the region, the more the individual’s education level, income, etc. affect aging. The stronger the impact of anxiety (Figure 1). Based on the above analysis proposed:

*Hypothesis* 5: The impact of individual socioeconomic status on aging anxiety is positively moderated by the level of marketization in their region.

## 3 Methodology

### 3.1 Data sources

This paper analyzes the problem of aging anxiety using data from the Chinese General Social Survey (CGSS) 2021, a national, comprehensive, and continuous academic survey organized by the China Center for Surveys and Data of Renmin University of China, which has been conducting a continuous cross-sectional survey of more than 10,000 households in all provinces, municipalities, and autonomous regions of mainland China once a year since 2003, covering various aspects of economic status and individual mindset. Since 2003, it has conducted continuous cross-sectional surveys on more than 10,000 households in all provinces and autonomous regions of mainland China. The data covers multiple levels of society, community, family, and individual, including various aspects of the economy, politics, society, culture, institutions, behavior, and attitudes, which provides relatively rich materials for researching the economic status as well as the individual’s mindset.

The year 2021 is the 14th annual survey of the China General Social Survey (CGSS), with a total of 8,148 valid samples completed nationwide. Core Module and Theme Module: the content of the module asks about all respondents, and the core module questions, which have been used since 2010, mainly contain socio-demographic attributes, housing, health, migration, lifestyle, social attitudes, class identity, labor market, social security, family, etc. Thematic modules include the combined impact of the New Crown epidemic, marriage and fertility intentions, and work and occupation. The additional health module of the EASS, which was answered by one-third of the randomly selected respondents, mainly covers health status, medical services, social trust, and concerns about aging. The health module of the East Asian Social Survey (EASS) is a repeat of the 2010 survey for this module and can be used for a longitudinal comparative study with 2010, as well as for a cross-sectional comparative study with Japan and South Korea of the EASS. The core variables for this study were drawn from the core module, the thematic module, and the East Asian Social Survey, and a total of 2,222 samples were extracted.

### 3.2 Variable operationalization

#### 3.2.1 Dependent variable

Drawing on the Anxiety about Aging Scale (AAS) developed by Kafer et al. (1980) and subsequent refinements by Lynch (2000), this study selected three specific items to operationalize the dependent variable, aging anxiety:

*“I worry that I will lose the ability to care for myself when I am old”* (termed self-care anxiety, reflecting concerns about physical mobility).

*“I worry that I will have to let others make decisions for me when I am old”* (termed autonomy anxiety, reflecting fears about diminished decision-making autonomy).

*“Financial dependence on others is one of my greatest concerns about aging”* (termed self-sufficiency anxiety, reflecting apprehensions about economic/financial independence).

Responses to all three items were recorded on a 5-point Likert scale (“*Strongly agree”* to “*Strongly disagree”*), reverse-coded (1–5) such that higher scores indicate greater anxiety.

To meet statistical analysis requirements, we conducted Exploratory Factor Analysis (EFA) on the three aforementioned items. The Kaiser-Meyer-Olkin (KMO) measure approached 0.7 (0.694), and Bartlett’s test of sphericity was significant (*p* < 0.001), confirming factor analysis appropriateness. Using principal component analysis, one factor with an eigenvalue greater than 1 was extracted, accounting for 68.01% of the total variance. Factor loadings for the three items were: Item 1 (0.820), Item 2 (0.844), Item 3 (0.810)—all exceeding 0.5, indicating they primarily reflect a common underlying factor. The factor scores were transformed into T-scores to represent aging anxiety scores. Cronbach’s *α* coefficient for the three items was 0.764, demonstrating satisfactory internal consistency reliability. Data limitations restricted measurement to only three aging anxiety items, potentially omitting other dimensions (e.g., fear of death, physical appearance changes, health decline). However, prior research (Lynch, 2000) indicates that in factor analyses of aging anxiety scales, health, physical functioning, and independence dimensions exhibit the highest factor loadings and contribute most substantially to overall model fit. Financial anxiety demonstrates secondary but significant loading contributions, while dimensions like physical appearance and social loss concerns contribute minimally. The current indicators—encompassing physical functioning, independence, and financial anxiety—though imperfect, collectively represent primary dimensions of aging anxiety.

#### 3.2.2 Independent variables

The independent variables primarily employ individuals’ educational attainment and income levels as proxies for socioeconomic status.

Educational Attainment: Assessed through the question: “*What is your highest level of education completed?”* Response options ranged from “*No formal education”* to “*Postgraduate degree or higher.”* These were coded numerically as follows:

1. *“No formal education,” “Private tutoring/literacy classes,” or “Primary school.”*
2. *“Junior high school.”*
3. *“Vocational high school,” “General high school,” “Secondary specialized school,” or “Technical school.”*
4. *“Junior college (adult higher education)” or “Junior college (formal higher education).”*
5. *“Bachelor’s degree (adult higher education)” or “Bachelor’s degree (formal higher education).”*
6. *“Postgraduate degree or higher.” Responses* labeled *“Other”* were excluded.

Personal income: Income level was measured using personal income, specifically assessed through the question: *“What was your total personal income in the previous year (2020)?”* To reduce data skewness and mitigate information loss, the natural logarithm of (reported income+1) was employed for analysis. Regarding the measurement of subjective socioeconomic status (SES), since it is substantially influenced not only by objective SES but also by psychological processes such as cultural context and social comparison, this study adopts a status mobility perspective. We operationalize the construct through expected change in subjective SES, derived from the differential between responses to two survey items:

1. *“Overall, which stratum of society do you* currently *occupy?”*
2. *“Which stratum do you expect to occupy* in *10 years?”*

Responses were recorded on a 10-point scale (1 = lowest, 10 = highest). Cases selecting *“do not know”* or “refused” were excluded. The resulting variable—subjective SES mobility expectation—was calculated as:

Expected Mobility = (Future Status Score)–(Current Status Score).

#### 3.2.3 Control variables

The control variables included basic demographic characteristics (age, age squared, gender, ethnicity, marital status, political affiliation, social insurance participation) alongside social support, social trust, and residential environment. The relationship between age and aging anxiety remains inconclusive: some studies identify an inverted U-shaped relationship (Lynch, 2000), while others observe declining anxiety with advancing age. Therefore, age squared was incorporated as a control variable. Additionally, empirical evidence indicates that neighborhood and household environments significantly impact older adults’ mental health (Sun and Sun, 2024). Consequently, we operationalized residential environment through six survey items:

1. *“My neighborhood (within 1 km/15-min walk) provides adequate facilities for physical exercise (*e.g.*, jogging, walking).”*
2. *“Sufficient public amenities are available in my residential area.”*
3. *“My neighborhood offers abundant access to fresh fruits and vegetables.”*
4. *“I perceive my residential area as safe.”*
5. *“Neighbors demonstrate mutual concern within my community.”*
6. *“Neighbors are willing to assist me when needed.”*

Responses were recorded on a 5-point Likert scale (1 = *completely disagree* to 5 = *completely agree*). The residential environment index was calculated as the mean score across these six items, with higher scores indicating better environmental conditions.

Social support was measured by the question: “*In the past year, has there usually been someone who listened to you when you needed to talk about matters of personal concern?”* Responses were dichotomized (“*Yes”* or “*No”*), with the option “*I have no matters of concern”* excluded.

Generalized trust was assessed using the item: “*Generally speaking, do you believe most people can be trusted, or do you think you need to be very careful in dealing with others?”* Response options included:” People *can almost always be trusted”* (4),"*People can often be trusted”* (3),"*You often need to be careful when dealing with others”* (2),"*You almost always need to be careful when dealing with others”* (1). Higher scores reflect greater trust, following reverse coding (4–1).

We measured social insurance participation using the *number of social insurance schemes* in which individuals are enrolled. The assessed categories include:

*“Urban Basic Medical Insurance / New Rural Cooperative Medical System / Commercial Health Insurance / Government Medical Insurance.”*

*“Urban/Rural Basic Pension Insurance.”*

*“Commercial Health Insurance.”*

*“Commercial Pension Insurance.”*

#### 3.2.4 Mechanism variable

The mediating variables include stressors and perceptions of aging. Stressors encompass both routine stressors (personal and familial) and anticipatory stressors.

##### 3.2.4.1 Routine stressors

Grounded in Pearlin’s Stress Process Theory, personal stressors involve negative life events and daily hassles that contribute to mental health outcomes (Pearlin, 1989). Empirical studies suggest that aging-related anxiety becomes more prevalent when older adults experience aging-associated adversities, such as chronic illnesses (Trigg et al., 2012). Additionally, perceptions of aging are influenced by factors like depression, health self-evaluations, and activity limitations (Janecková et al., 2013).

##### 3.2.4.2 Personal stressors

Measured by the question: “*Do you have any chronic diseases or long-term health conditions?”* (Yes/No).

##### 3.2.4.3 Familial stressors

Assessed via: “*Among your cohabiting or non-cohabiting family members, is there anyone requiring long-term care due to chronic physical/mental illness, disability, or frailty associated with aging?”* (Yes/No).

##### 3.2.4.4 Anticipatory stressors

Distinct from immediate, tangible stressors, anticipatory stressors reflect perceived future threats rather than current realities (Pearlin and Bierman, 2013). Accordingly, this study operationalizes two types of anticipated stressors:

###### 3.2.4.4.1 Public health emergency stressor (anticipated stressor I)

Measured by: *“How likely do you think you are to contract COVID-19?”*

Response scale: *“Very likely”* to *“Very unlikely”* (7-point scale).

Scoring: Reverse-coded as 7–1 for analytical convenience.

###### 3.2.4.4.2 Healthcare access stressor (anticipated stressor II)

Measured by: *“Are you worried that ‘you or your family may be unable to access medical services when needed’?”*

Response scale: *“Very worried” to* “Not worried at all” (4-point scale).

Scoring: Reverse-coded as 4–1 for analytical convenience.

##### 3.2.4.5 Perceptions of aging

Measured by the item: “*Do you agree that older adults are a burden to society?”* Responses included “*Strongly agree”* (1), “*Agree”* (2), “*Disagree”* (3), and “*Strongly disagree”* (4), with higher scores indicating more positive perceptions.

##### 3.2.4.6 Regional marketization level

Regional marketization level was included as a moderating variable, operationalized using the province-level marketization index developed by Professor Fan Gang’s research team at Peking University.

### 3.3 Methods of analysis

Given the nested structure of the data, where the impact of socioeconomic status on aging anxiety may be moderated by province-level contextual factors, this study employs hierarchical linear modeling (HLM). Province-level variables were modeled at Level 2 to examine both the direct effects of socioeconomic status on overall aging anxiety and the moderating role of marketization level. To analyze the mediating mechanisms through which socioeconomic status influences aging anxiety, structural equation modeling (SEM) was utilized. Mediation tests were conducted using the Baron and Kenny (1986) approach and the Zhao et al. (2010) procedure to quantify indirect effects of socioeconomic status indicators on the dependent variable via mediating variables. Given the complex relationship between objective/subjective socioeconomic status (SES) and aging anxiety, we employed structural equation modeling (SEM) to analyze the mechanism whereby objective SES may influence aging anxiety through the mediating effect of subjective status mobility expectations. For heterogeneity analysis considering the ordinal nature of the three domain-specific aging anxiety variables (five-category ordinal variables), we implemented hierarchical ordered probit (Oprobit) regression to examine SES effects on each specific anxiety dimension (Table 1).

**Table 1:** Descriptive statistics of variables.

VarName	Mean	SD	Min	Max
Aginganxiety_T	50.000	10.000	24.924	67.138
(a person’s) Age	51.644	17.574	18.000	99.000
Age squared (Age*Age/100)	29.759	17.999	3.240	98.010
Gender (female = 0)	0.452	0.498	0.000	1.000
Ethnicity (minority = 0)	0.926	0.261	0.000	1.000
Marital status (unmarried = 0)	0.711	0.453	0.000	1.000
Political affiliation (non-party members = 0)	0.119	0.324	0.000	1.000
Social support	0.558	0.497	0.000	1.000
General trust	2.844	0.750	1.000	4.000
Residential environment	2.127	0.558	1.000	4.500
Social insurance	1.851	0.781	0.000	4.000
Educational attainment	1.411	1.382	0.000	5.000
Personal income (natural logarithmic)	8.074	4.217	0.000	12.794
Status mobility	0.829	1.540	−9.000	9.000
Family stressors	0.254	0.435	0.000	1.000
Anticipatory stressors I	2.396	1.612	1.000	7.000
Anticipatory stressors II	2.214	1.026	1.000	4.000
Personal stressors	0.378	0.485	0.000	1.000
Perceptions of aging	2.927	0.759	1.000	4.000

## 4 Results of empirical analysis

### 4.1 Status and changes in aging anxiety

As shown in Table 2, the overall mean aging anxiety score^1^ decreased marginally from 10.212 in 2010 to 10.126 in 2021. Specifically: the mean anxiety about physical mobility declined from 3.656 to 3.637; anxiety regarding decision-making autonomy increased slightly from 3.260 to 3.265; while anxiety concerning financial independence decreased most substantially from 3.296 to 3.224. Independent samples t-tests indicated non-significant changes for overall aging anxiety (*p* = 0.108), physical mobility anxiety (*p* = 0.156), and decision-making autonomy anxiety (*p* = 0.964). However, financial independence anxiety showed a statistically significant decline at 95% CI (*p* = 0.008). These patterns suggest that improved income levels accompanying socioeconomic development have exerted a mitigating effect on financial anxiety about aging.

**Table 2:** Comparative changes in aging anxiety.

Year	Self-care anxiety	Autonomy anxiety	Self-sufficiency anxiety	Aging anxiety
2010	3.656	3.260	3.296	10.212
2021	3.637	3.265	3.224	10.126

### 4.2 Impact of socioeconomic status on aging anxiety

Adding the socio-demographic variables, social support and social trust variables as a baseline regression model in Model 1 of Table 3 shows that both age and age squared have a significant effect on aging anxiety, with an inverted U-shaped relationship between age and aging anxiety. Gender significantly influences aging anxiety, with males reporting lower levels than females. Ethnicity and marital status show no significant association with aging anxiety. Communist Party members exhibit significantly less aging anxiety compared to non-members. Social support, generalized trust, and social insurance participation demonstrate significant effects on aging anxiety: higher trust in others correlates with lower aging anxiety; greater social support corresponds to reduced aging anxiety; enrollment in more social insurance schemes predicts decreased anxiety. Notably, we observe a paradoxical pattern where increased social support is associated with higher aging anxiety—contradicting prior research (Ramírez and Palacios-Espinosa, 2016). This anomaly warrants verification through subsequent regression analysis.

**Table 3:** Hierarchical linear regression analysis of the impact of socioeconomic status on aging anxiety.

Predictors	Aging anxiety				
	Model 1	Model 2	Model 3	Model 4	Model 5
Age	0.285^***^ (0.070)	0.226^**^ (0.085)	0.168^*^ (0.080)	0.209^*^ (0.085)	0.154^+^ (0.080)
Age squared	−0.269^***^ (0.067)	−0.244^**^ (0.079)	−0.187^*^ (0.074)	−0.239^**^ (0.079)	−0.183^*^ (0.074)
Gender	−1.720^***^ (0.382)	−1.238^**^ (0.427)	−0.676^+^ (0.401)	−1.216^**^ (0.425)	−0.658^+^ (0.400)
Ethnicity	−0.848 (0.826)	−0.198 (0.900)	0.866 (0.838)	−0.186 (0.895)	0.871 (0.835)
Marital status	−0.011 (0.481)	0.083 (0.527)	0.091 (0.495)	0.149 (0.525)	0.149 (0.493)
Political affiliation	−1.280^*^ (0.590)	−0.599 (0.667)	0.176 (0.627)	−0.560 (0.664)	0.210 (0.624)
Social support	1.279^**^ (0.396)	1.198^**^ (0.437)	0.563 (0.411)	1.312^**^ (0.435)	0.674^+^ (0.410)
General trust	−0.915^***^ (0.253)	−0.844^**^ (0.277)	−0.695^**^ (0.260)	−0.754^**^ (0.277)	−0.614^*^ (0.260)
Residential environment	0.360 (0.341)	0.629^+^ (0.374)	−0.178 (0.353)	0.623^+^ (0.372)	−0.179 (0.352)
Social insurance	−0.819^**^ (0.253)	−0.663^*^ (0.277)	−0.469^+^ (0.259)	−0.625^*^ (0.276)	−0.436^+^ (0.258)
Educational attainment		−0.578^**^ (0.210)	−0.411^*^ (0.197)	−0.551^**^ (0.210)	−0.388^*^ (0.196)
Personal income (natural logarithmic)		−0.115^*^ (0.054)	−0.111^*^ (0.051)	−0.101^+^ (0.054)	−0.099^+^ (0.051)
Status mobility		−0.310^*^ (0.143)	−0.330^*^ (0.134)	−0.325^*^ (0.142)	−0.344^*^ (0.134)
Family stressors			1.646^***^ (0.446)		1.630^***^ (0.444)
Anticipatory stressors I			0.757^***^ (0.119)		0.747^***^ (0.118)
Anticipatory stressors II			2.852^***^ (0.199)		2.850^***^ (0.198)
Personal stressors			1.340^**^ (0.453)		1.260^**^ (0.451)
Perceptions of aging				−1.268^***^ (0.282)	−1.156^***^ (0.265)
_cons	47.776^***^ (2.143)	50.168^***^ (2.625)	54.561^***^ (2.511)	53.971^***^ (2.746)	58.036^***^ (2.624)
lns1_1_1_cons	0.473^*^ (0.199)	0.375^+^ (0.216)	0.246 (0.225)	0.368^+^ (0.217)	0.242 (0.226)
lnsig_e_cons	2.272^***^ (0.014)	2.257^***^ (0.015)	2.192^***^ (0.015)	2.253^***^ (0.015)	2.188^***^ (0.015)
N	2,713	2,222	2,222	2,222	2,222

(1) Standard errors in parentheses; (2) + *p* < 0.1, *p < 0.05, **p < 0.01, ***p < 0.001.

Model 2 introduces socioeconomic status variables to Model 1. While controlling for other variables, educational attainment demonstrates a significant negative effect on aging anxiety: per one-level increase in education, aging anxiety scores decrease by 0.578 units. Personal income exhibits a significant negative effect on aging anxiety: with every 1% increase in income, aging anxiety scores decrease by 11.5%. Subjective socioeconomic status mobility expectations show a significant negative effect on aging anxiety: per one-level increase in anticipated status mobility, aging anxiety scores decrease by 31%.

Model 3 expands upon Model 2 by incorporating familial, personal, and anticipated stressors potentially influencing aging anxiety. Family stressors demonstrate a significant positive effect: while controlling for other variables, individuals experiencing family stressors exhibit aging anxiety scores 1.646 units higher than those without such stressors. When family members require care due to chronic physical/mental illnesses, disabilities, or age-related frailty, individuals face significantly elevated aging anxiety—indicating stress proliferation mechanisms at work. Personal stressors significantly increase aging anxiety: individuals with chronic illnesses or persistent health problems show anxiety scores 1.340 units higher than unaffected counterparts after controlling for covariates. Anticipated stressors also exert significant positive effects: Each unit increase in fears regarding public health emergencies (e.g., COVID-19) corresponds to a 0.757-unit increase in aging anxiety scores; Each unit decrease in anticipated healthcare access barriers predicts a 2.852-unit decrease in aging anxiety scores; Concerns about disasters like COVID-19 significantly heighten aging anxiety.

Meanwhile, when the stressor-related variables were added to model 3, the significance of the effect of social support on aging anxiety decreased, indicating that social support may be affected by stressors. As for the measure of social support, “Do people usually listen to you about your concerns?,” respondents who choose “Yes” give the most feedback to the two types of listeners: “family members who live with you” (58.026% of the effective responses) and “friends” (55.132% of the effective responses). According to the stress process theory, stressors will spread. Family members living with illness or needing care may hurt the listening effect. According to the contact theory, only quality contact can reduce aging anxiety, so listening to family members may hurt aging anxiety. While friends are less likely to be our caregivers (Barrett and Lynch, 1999), the quality of relationships with friends may not affect concerns about declining health in old age (Barrett and Robbins, 2008), and listening from friends may not have an effect on aging anxiety, so social support, as measured by listening, may not play a debilitating role in aging anxiety.

Model 4 adds the variable of the individual’s perception of the elderly based on model 2, and it can be seen that an individual’s perception of the elderly has a significant negative effect on aging anxiety, and for every unit increase in the degree of an individual’s positive perception of the elderly, their aging anxiety decreases by 1.268 units, and the more the individual disagrees with the statement of *“the elderly are a burden to society,”* the less their aging anxiety is. The more an individual disagrees with the statement *“the elderly are a burden on society,”* the less aging anxiety he or she has.

Model 5 represents the full model incorporating all variables from previous models. Core variables—socioeconomic status, stressors, and aging perceptions—maintain significant effects on aging anxiety, confirming Hypotheses 2.1, 1.1, and 1.3. However, ethnicity, marital status, and political affiliation become non-significant in this model, likely due to effect dilution by other covariates. Age squared retains significant influence, indicating an inverted U-shaped relationship between age and aging anxiety. U-test verification (Table 4) reveals an inflection point at approximately 42 years within the observed age range [18, 99]. The slope pattern (positive before inflection, negative after) confirms this curvature. This aligns with the “aging paradox”: while society often associates aging with decline, older adults frequently perceive it as an opportunity for growth and positive transformation. Early old age may bring increased well-being and life satisfaction (Schilling, 2006), with diminishing negative age effects. These findings correspond with Lynch (2000) conclusion that aging anxiety peaks between ages 40–50.

**Table 4:** U-shaped relationship test between age and aging anxiety.

Metrics	Lower bound	Upper bound
Interval	18	99
Slope	0.086	−0.197
*t*-value	1.586	−2.761
P > t	0.056	0.003
Extreme point	42.670	

### 4.3 Mediating role of anxiety stressors and perceptions of aging

After introducing familial, personal, and anticipated stressors in Model 3 (Table 3), educational attainment, income, and status mobility maintained significant effects on aging anxiety. However, both significance levels and regression coefficients decreased substantially. These stressors likely function as mediating variables between socioeconomic status variables and aging anxiety—their inclusion attenuated the influence of socioeconomic status through mediating effects.

When aging perception variables were added in Model 4 (Table 3), the significant effects of education and income persisted but with diminished significance and coefficients. This likely occurs because aging perceptions dilute the impact of education and income on aging anxiety, acting as mediators.

To further test whether socioeconomic status influences aging anxiety through anxiety stressors and perceptions of old age, the mediating roles of anxiety stressors and perceptions of aging were analyzed through structural equation modeling.

Structural equation modeling mediation analysis presented in Table 5 demonstrates that, after controlling for covariates, educational attainment exerts significant negative effects on family stressors, personal stressors, and anticipated healthcare access barriers while simultaneously generating significant positive effects on aging perceptions. Higher educational levels correspond to reduced exposure to family- and personally-related aging anxiety stressors and more favorable perceptions of aging. Mediation analysis confirms that education partially influences aging anxiety through these pathways: family stressors, personal stressors, healthcare access barriers, and aging perceptions. Personal income shows significant negative effects on personal aging-related stressors and significant positive effects on aging perceptions, though no significant impact on family stressors. Elevated income levels predict diminished personal stressors and more positive aging perceptions. Mediation testing verifies that income affects aging anxiety indirectly through personal stressors and aging perceptions. Subjective socioeconomic status mobility expectations negatively impact both personal stressors and anticipated stressors, suppressing health-related and prospective pressures. Empirical mediation pathways confirm that subjective mobility expectations influence aging anxiety through these stressor channels, thereby validating Hypotheses 3 and 4.

**Table 5:** Analysis of mediating effects of structural equation modeling.

Predictors	Family stressors	Personal stressors	Anticipatory stressors I	Anticipatory stressors II	Perception of aging	Status mobility
	Model 1	Model 2	Model 3	Model 4	Model 5	Model 6
Educational attainment	−0.016^*^ (0.007)	−0.092^***^ (0.007)	−0.004 (0.025)	−0.032^*^ (0.016)	0.095^***^ (0.010)	0.175^***^ (0.024)
Mediating effects test	Partial mediation	Partial mediation	No mediation	Partial mediation	Partial mediation	Partial mediation
Personal income	−0.000 (0.002)	−0.005^*^ (0.002)	−0.002 (0.008)	−0.004 (0.005)	0.010^**^ (0.004)	−0.02^**^ (0.008)
Mediating effects test	No mediation	No mediation	No mediation	No mediation	Partial mediation	Partial mediation
Status mobility	−0.009 (0.007)	−0.031^***^ (0.007)	−0.048^*^ (0.023)	−0.039^*^ (0.015)	0.010 (0.011)	
Mediating effects test	No mediation	Partial mediation	Partial mediation	Partial mediation	No mediation	
Personal stressors					−0.069^+^ (0.036)	
Control variables	———	———	———	———	———	———
_cons	0.277^***^ (0.021)	0.564^***^ (0.023)	2.448^***^ (0.082)	2.145^***^ (0.050)	3.009^***^ (0.199)	0.732^***^ (0.071)

Due to space constraints, information related to other independent, control and dependent variables is not listed in the table. (1) Standard errors in parentheses; (2) + *p* < 0.1, *p < 0.05, **p < 0.01, ***p < 0.001.

Notably, chronic illness stressors significantly undermine rational aging perceptions after covariate adjustment, indicating that prolonged stress exposure may erode cognitive frameworks regarding aging. Model 6 in Table 5 further reveals that objective socioeconomic status indicators (education and income) exert indirect effects on aging anxiety through subjective SES mobility expectations. Approximately 13% of education’s total effect and 7% of income’s total effect operate via this subjective expectation pathway. This evidence establishes that while objective and subjective SES independently influence aging anxiety, objective status also operates indirectly through subjective mobility expectations’ mediating role.

### 4.4 The moderating effect of regional marketization level

Table 6 examines whether provincial marketization levels moderate the relationship between socioeconomic status and aging anxiety. Analysis reveals that while the anxiety-reducing effects of personal income and subjective socioeconomic status mobility expectations show positive moderation by marketization levels, this moderation is statistically non-significant. Conversely, educational attainment’s mitigating effect on aging anxiety demonstrates significant positive moderation by regional marketization: the higher a province’s marketization level, the stronger education’s inhibitory effect on aging anxiety. Hypothesis 5 is thus partially supported.

**Table 6:** Analysis of the moderating effects of regional marketization level.

Predictors	Aging anxiety	Aging anxiety	Aging anxiety
	Model 1	Model 2	Model 3
Educational attainment	−0.392^*^ (0.196)	−0.390^*^ (0.196)	1.662^+^ (0.951)
Personal income	0.093 (0.324)	−0.099^+^ (0.051)	−0.099^+^ (0.051)
Status mobility	−0.341^*^ (0.134)	0.114 (0.759)	−0.347^**^ (0.134)
Marketization level	0.244 (0.377) (0.115)	0.121 (0.254) (0.081)	0.403 (0.280) (0.089)
Personal income*Personal income	−0.021 (0.035)		
Status mobility*Marketization level		−0.052 (0.085)	
Educational attainment*Marketization level			−0.226^*^ (0.102)
Control variables	———	———	———
_cons	55.931^***^ (4.149)	56.979^***^ (3.343)	54.384^***^ (3.514)

Due to space constraints, information related to other independent, control and dependent variables is not listed in the table. (1) Standard errors in parentheses; (2) + *p* < 0.1, *p < 0.05, **p < 0.01, ***p < 0.001.

### 4.5 Robustness test and heterogeneity analysis

To address potential endogeneity arising from reverse causality (where aging anxiety may influence socioeconomic status), Table 7 implements instrumental variable (IV) analysis. Grounded in life course theory, early-life family circumstances may shape later development. Accordingly: Model 1 employs the mother’s *Hukou status*^2^ (rural/urban) at the respondent’s birth as an IV for educational attainment, conducting two-stage least squares (2SLS) estimation to test the robustness of education’s effect on aging anxiety. Model 2 results demonstrate that education retains a significant negative effect on aging anxiety after covariate adjustment: higher education predicts lower anxiety scores. Model 3 incorporates the father’s employment status when the respondent was age 14 as an IV for current income. Model 4 results confirm income’s persistent significant negative effect on aging anxiety after controlling for confounders.

**Table 7:** Educational attainment, personal income and aging anxiety(Robustness test).

Predictors	Educational attainment	Aging anxiety	Personal income	Aging anxiety
	Model 1	Model 2 (2SLS)	Model 3	Model 4 (2SLS)
Aging anxiety		−1.473^*^ (0.618)		
Personal income				−1.299^*^ (0.482)
Mother’s Hukou	−0.748^***^ (0.046)			
Father’s Occupation			1.079^***^ (0.187)	
Control variables	———	———	———	———
_cons	4.703^***^ (0.227)	54.528^***^ (3.519)	−1.120^***^ (1.015)	46.639^***^ (2.756)
N	2,411	2,411	2,106	2,106

Due to space constraints, information related to other independent, control and dependent variables is not listed in the table. (1) Standard errors in parentheses; (2) + *p* < 0.1, *p < 0.05, **p < 0.01, ***p < 0.001.

Model 1 in Table 8 substitutes the original 1–12 coding of educational attainment with the share of personal income in household income (natural logarithm transformed). Regression analysis reveals that: A higher share of personal income in household income exerts a significant negative effect on aging anxiety. This income share measure reflects economic independence: greater independence potentially enhances future risk management capacity (Barrett and Robbins, 2008), thereby reducing aging anxiety.

**Table 8:** Robustness test of the mechanism of the effect of socioeconomic status on aging anxiety.

Predictors	Aging anxiety	Self-care anxiety	Autonomy anxiety	Self-sufficiency anxiety	Aging anxiety (mean)
	M1 (HLM)	M2 (oprobit)	M3 (oprobit)	M4 (oprobit)	M5 (Oprobit)
Educational attainment	−0.501^*^ (0.212)	0.001 (0.024)	−0.019 (0.023)	−0.095^***^ (0.023)	−0.047^*^ (0.022)
Personal income		−0.011^+^ (0.006)	−0.007 (0.006)	−0.015^*^ (0.006)	−0.012^*^ (0.006)
Status mobility	−0.319^*^ (0.147)	−0.054^***^ (0.016)	−0.018 (0.016)	−0.032^*^ (0.016)	−0.037^*^ (0.015)
Familial stressors	1.298^**^ (0.489)	0.257^***^ (0.055)	0.147^**^ (0.053)	0.112^*^ (0.053)	0.180^***^ (0.051)
Anticipatory stressors I	0.741^***^ (0.130)	0.069^***^ (0.015)	0.076^***^ (0.014)	0.062^***^ (0.014)	0.084^***^ (0.014)
Anticipatory stressors II	2.830^***^ (0.220)	0.321^***^ (0.025)	0.238^***^ (0.024)	0.250^***^ (0.024)	0.320^***^ (0.023)
Personal stressors	0.979^+^ (0.500)	0.217^***^ (0.055)	0.066 (0.054)	0.075 (0.054)	0.147^**^ (0.052)
Perceptions of aging	−1.252^***^ (0.297)	−0.089^**^ (0.033)	−0.137^***^ (0.032)	−0.148^***^ (0.032)	−0.139^***^ (0.030)
Personal income/household income	−1.209^+^ (0.689)				
Control variables	———	———	———	———	———
_cons	44.334^***^ (3.012)				
lns1_1_1_cons	0.280 (0.233)				
lnsig_e_cons	2.188^***^ (0.017)				
N	1805	2,222	2,222	2,222	2,222

Due to space constraints, information related to other independent, control and dependent variables is not listed in the table. (1) Standard errors in parentheses; (2) + *p* < 0.1, *p < 0.05, **p < 0.01, ***p < 0.001.

Table 8 presents domain-specific analyses examining distinct dimensions of aging anxiety. Model 2 employs anxiety about physical mobility as the dependent variable, Model 3 anxiety regarding decision-making autonomy, and Model 4 anxiety concerning financial independence. Model 5 utilizes a composite aging anxiety measure derived from the mean score of these three dimensions. Given the ordinal nature of these dependent variables, hierarchical ordered probit regression was implemented. Results from Model 6 demonstrate substantial consistency with Table 3 Model 5, with key explanatory variables maintaining significant effects on aging anxiety. Domain-specific patterns reveal differential effects: personal income exhibits non-significant effects on autonomy anxiety but significant negative effects on financial independence anxiety. For physical mobility anxiety, a negative effect emerges at the 10% significance level. Income’s aggregate influence primarily manifests through financial independence and physical mobility dimensions. Subjective socioeconomic status mobility expectations significantly reduce physical mobility anxiety, with stronger expectations predicting lower anxiety about age-related physical dependence. While demonstrating non-significant effects on autonomy anxiety, these expectations significantly reduce financial independence anxiety at the 5% significance level. Thus, subjective mobility expectations predominantly influence composite anxiety through physical functioning and financial security pathways. Educational attainment maintains significant negative effects on composite anxiety and specifically on financial independence anxiety, yet shows non-significant effects on physical mobility and autonomy anxieties. Education’s aggregate impact is manifested primarily through mitigating concerns about declining financial independence during aging.

Table 9 presents subgroup analyses stratified by urban–rural residence. Regression results indicate that while stressors and aging perceptions significantly influence aging anxiety for both groups after covariate adjustment, educational attainment and personal income show non-significant effects on aging anxiety among rural residents—consistent with existing research (Zimmer and Amornsirisomboon, 2001). Subjective socioeconomic status mobility expectations negatively affect urban residents’ anxiety at the 10% significance level and rural residents’ anxiety at the 5% level. This suggests that objective socioeconomic status primarily influences urban populations, whereas subjective status exerts stronger effects among rural residents. This divergence likely relates to differential intra-regional stratification: educational and income disparities may be less pronounced among rural residents compared to urban counterparts. Urban areas offer greater diversity and quantity of aging-support resources (e.g., healthcare, pensions); higher objective SES enables access to more anti-aging resources, thereby reducing anxiety. Conversely, limited availability of aging-support services in rural areas objectively constrains preparatory opportunities. Subjective status expectations provide psychological motivation and perceived future control, mitigating anxiety.

**Table 9:** Differences in aging anxiety between urban and rural residents.

Predictors	Urban Aging Anxiety	Rural aging anxiety	General aging anxiety
	Model 1	Model 2	Model 3
Educational attainment	−0.148^*^ (0.064)	−0.046 (0.127)	−0.128^*^ (0.057)
Personal income	−0.038^*^ (0.018)	−0.008 (0.026)	−0.029^*^ (0.015)
Status mobility	−0.082^+^ (0.048)	−0.135^*^ (0.062)	−0.098^**^ (0.038)
Family stressors	0.484^**^ (0.157)	0.454^*^ (0.213)	0.466^***^ (0.126)
Anticipatory stressors I	0.176^***^ (0.040)	0.278^***^ (0.060)	0.209^***^ (0.034)
Anticipatory stressors II	−0.839^***^ (0.068)	−0.768^***^ (0.101)	−0.813^***^ (0.056)
Personal stressors	0.357^*^ (0.158)	0.466^*^ (0.222)	0.367^**^ (0.128)
Perceptions of aging	−0.297^**^ (0.095)	−0.381^**^ (0.123)	−0.332^***^ (0.075)
Urban–rural			0.503 (0.312)
Social insurance	−0.062 (0.084)	−0.359^*^ (0.150)	0.240 (0.216)
Urban–rural#social insurance			−0.293^+^ (0.163)
Control variables	*———*	*———*	*———*
_cons	12.293^***^ (0.915)	12.851^***^ (1.350)	11.770^***^ (0.814)
lns1_1_1_cons	−0.988^***^ (0.258)	−0.956^**^ (0.371)	−1.021^***^ (0.225)
lnsig_e_cons	0.911^***^ (0.018)	0.946^***^ (0.026)	0.928^***^ (0.015)
N	1,487	735	2,222

Due to space constraints, information related to other independent, control and dependent variables is not listed in the table. (1) Standard errors in parentheses; (2) + *p* < 0.1, *p < 0.05, **p < 0.01, ***p < 0.001.

Mann–Whitney U tests confirm significantly higher aging anxiety scores among rural residents (mean = 10.411) versus urban residents (mean = 9.982; *p* < 0.001). Model 3 (Table 9) incorporating an urban–rural × social insurance interaction term reveals that increased social insurance enrollment significantly moderates rural–urban anxiety disparities, particularly reducing anxiety in rural populations. Social insurance functions as an institutional safety net, effectively alleviating survival uncertainty among rural residents. These findings collectively demonstrate that heightened aging anxiety in rural populations stems partially from relative deficiencies in social insurance coverage compared to urban areas, exacerbating concerns about elderly livelihood security.

## 5 Conclusion and discussion

### 5.1 Conclusion

This study integrates the Fundamental Cause Theory and Stress Process Theory to analyze the impact of socioeconomic status on aging anxiety and its mechanisms, utilizing data from the 2021 Chinese General Social Survey (CGSS2021). Key findings reveal a modest decline in financial independence anxiety compared to 2010. Socioeconomic status significantly influences aging anxiety through both direct effects and indirect pathways mediated by stressors and aging perceptions. Educational attainment demonstrates significant anxiety-reducing effects: higher education correlates with reduced exposure to familial and personal stressors (e.g., health and caregiving challenges), diminished healthcare access concerns, and more positive aging perceptions, collectively lowering anxiety levels. Personal income exerts significant negative effects on aging anxiety, with elevated income predicting decreased probability of chronic disease exposure, enhanced positive aging perceptions, and consequent anxiety reduction. Subjective socioeconomic status mobility expectations significantly inhibit aging anxiety. Increased expectations correlate negatively with both immediate stressors and anticipated pressures. Furthermore, objective socioeconomic status indirectly influences aging anxiety through subjective mobility expectations, while stress exposure simultaneously undermines constructive aging perceptions.

Composite aging anxiety manifests through domain-specific concerns: physical mobility anxiety (self-care anxiety), decision-making autonomy anxiety (autonomy anxiety), and financial independence anxiety (self-sufficiency anxiety). Socioeconomic status influences these dimensions differentially. Personal income and subjective status mobility expectations primarily affect composite anxiety through self-sufficiency and self-care anxieties. Educational attainment significantly reduces self-sufficiency anxiety but shows non-significant effects on self-care and autonomy anxieties, exerting its aggregate influence predominantly via economic and financial independence concerns during aging.

Urban–rural structural transition and marketization constitute the macro-contextual backdrop for aging anxiety in China. The anxiety-reducing effects of socioeconomic status are moderated by urban–rural location and urbanization levels. Rural residents exhibit significantly higher aging anxiety than urban counterparts, partially attributable to disparities in social insurance coverage. Increased insurance enrollment moderates this urban–rural anxiety gap, particularly alleviating rural anxiety through institutional safety nets. Objective socioeconomic status (education/income) significantly reduces aging anxiety only among urban residents, demonstrating non-significant effects in rural areas. Conversely, subjective status mobility expectations more potently inhibit anxiety in rural populations. Higher regional urbanization strengthens education’s mitigating effect on aging anxiety, with urbanized contexts amplifying education’s protective role.

### 5.2 Discussion and policy implications

The Stress Process Theory posits that social status determines individuals’ exposure to stressors, which interact with psychological and social resources to shape mental health outcomes. However, existing research primarily focuses on quotidian and episodic stressors, leaving anticipated stressors understudied (Pearlin and Bierman, 2013). This study extends the theory empirically by introducing anticipated stressors and examining how socioeconomic status influences aging anxiety through this pathway. Furthermore, our analysis incorporates macro-structural dimensions—urban–rural disparities and regional marketization levels—to explore contextual moderation effects, thereby advancing theoretical understanding of market transition dynamics.

From a health sociology perspective, socioeconomic status influences health through four primary pathways: neo-materialist, psychosocial, cultural/behavioral, and life-course perspectives (Cockerham, 2017). This study adopts the neo-materialist lens—represented by the Fundamental Cause Theory—while integrating psychosocial elements (subjective SES mobility expectations, aging perceptions, immediate and anticipated stressors) to analyze aging anxiety. We further examine the moderating role of macro-level urban–rural structures in the SES-anxiety relationship. Future research should explore cultural/behavioral and life-course perspectives to deepen understanding of SES-aging anxiety mechanisms. Although this study advances existing literature by examining composite aging anxiety and its specific dimensions (physical mobility, decision-making autonomy, financial independence), it insufficiently addresses anxiety facets like physical appearance concerns, social status loss, and death anxiety due to data limitations. Similarly, our operationalization of anticipated stressors remains narrow, potentially failing to capture the full spectrum theorized in the Stress Process Framework. More comprehensive indicators and data are needed. The cross-sectional nature of our data precludes definitive causal inferences, as reverse causality and omitted variable bias cannot be fully excluded. Future investigations should employ longitudinal designs, natural experiments, or intervention studies to rigorously identify causal mechanisms. Internationally, surveys like the East Asian Social Survey have accumulated comparable data on aging anxiety. Cross-cultural comparative research represents a critical frontier for expanding the breadth and depth of this field.

The analysis of aging anxiety yields critical policy implications: First, sustained efforts to advance high-quality economic and social development must prioritize equitable distribution of developmental benefits across urban and rural populations. This requires deepening rural revitalization initiatives to enhance farmers’ confidence in future well-being and foster proactive attitudes toward aging-related challenges. Second, cultivating a society that respects and values the elderly is imperative. Leveraging both online and offline platforms, diverse public awareness campaigns should promote accurate understandings of aging, combat age-based discrimination and negative stereotypes, and encourage adaptive responses to age-related life transitions. Third, health risks within families, caregiving stressors, and anticipated healthcare access barriers significantly contribute to aging anxiety. Therefore, enhancing institutional arrangements for social security systems—particularly family and medical insurance—is imperative. We recommend advancing long-term care systems while prioritizing support for rural residents and low-income vulnerable groups. Such institutional safeguards will mitigate aging-related concerns by addressing fundamental security gaps.

## Data Availability

The dataset associated with this article is not publicly accessible due to restrictions on the original data. The raw data used in this study were obtained from the Chinese General Social Survey (CGSS), administered by the China Survey and Data Center at Renmin University of China. Access to this dataset was granted to the authors through formal authorization. Researchers interested in accessing the data may register and submit a request via the official platform: https://www.cnsda.org/index.php?r=projects/view&id=65635422 (Contact email: nsrc@ruc.edu.cn), or contact the corresponding author of this article for assistance: SY: xingzaijianghu@126.com.

## Footnotes

^1^To visualize temporal trends in aging anxiety between 2010 and 2021, responses to the three domain-specific aging anxiety items were summed to construct a composite aging anxiety variable, maintaining analytical consistency when comparing overall and specific anxiety dimensions.

^2^Hukou is the internationally recognized academic term for China’s household registration system (used in journals like Demography, China Quarterly).Status specifies the categorical nature (e.g., agricultural/non-agricultural, location).
